# Goals of Frail Older People Living With Chronic Kidney Disease: A Mixed Methods Study

**DOI:** 10.1111/jgs.19421

**Published:** 2025-03-05

**Authors:** Benignus Logan, Kristiana Ludlow, Elaine M. Pascoe, Andrea K. Viecelli, David W. Johnson, Carmel M. Hawley, Laura E. Hickey, Charani Kiriwandeniya, Misa Matsuyama, Allison Jaure, Ruth E. Hubbard

**Affiliations:** ^1^ Australian Frailty Network The University of Queensland Brisbane Australia; ^2^ Centre for Health Services Research The University of Queensland Brisbane Australia; ^3^ Department of Medicine Mater Hospital Brisbane Australia; ^4^ Internal Medicine Services, The Prince Charles Hospital Brisbane Australia; ^5^ Australasian Kidney Trials Network The University of Queensland Brisbane Australia; ^6^ Department of Kidney and Transplant Services Princess Alexandra Hospital Brisbane Australia; ^7^ Centre for Kidney Disease Research Translational Research Institute Brisbane Australia; ^8^ Sydney School of Public Health The University of Sydney Sydney Australia; ^9^ Department of General and Geriatric Medicine Princess Alexandra Hospital Brisbane Australia

**Keywords:** chronic kidney disease, frailty, goal attainment scaling, older people, quality of life

## Abstract

**Background:**

Frail older adults with chronic kidney disease (CKD) have complex care needs, and their priorities may differ from those assumed by healthcare providers. Understanding their goals is crucial to delivering person‐centred care. This study aimed to identify and categorize the goals of this population and determine any association with participants' frailty status, quality of life, and CKD stage.

**Methods:**

We report the goals of frail older people living with moderate to severe CKD enrolled as participants in the GOAL trial, a cluster‐randomized controlled trial assessing the effectiveness of comprehensive geriatric assessment. This study employs a mixed‐methods approach, utilizing a triangulation design and a data transformation model. Participants set goals by Goal Attainment Scaling. Deductive content analysis was undertaken, aided by a pre‐specified matrix (physical health; psychological health; function; planning; social engagement). Descriptive statistics assessed the relationships between goals and participant characteristics.

**Results:**

The 224 participants (mean age 77 [±6.7]; 56% male; 84% white/European; median FI 0.39 [IQR: 0.33–0.47]) set 408 goals in the categories of function, physical health, social engagement and leisure, psychological health, and future readiness. Most participants set one or two goals (*n* = 183, 82%). They were most frequently set in the function (*n* = 172, 42%), physical health (*n* = 86, 21%), and social engagement and leisure (*n* = 79, 19%) domains. The number and nature of the set goals were similar across participant frailty status, quality of life (EQ‐5D‐5L) scores, and CKD stage.

**Conclusion:**

Frail older adults with CKD most frequently focus their goals on function, physical health, social engagement, and leisure. These goals did not vary by participants' frailty status or CKD stage. This study's findings can guide healthcare professionals in ensuring management plans consider these identified priorities. Geriatricians may have a role in managing this population, given the commonality of these goals with those of older people more generally.

**Summary**

- Key points
  ○ Prior research shows that people living with CKD identified kidney function, kidney failure, fatigue, mortality, and life participation as critically important outcomes, while older people highlighted some specific priorities including maintaining independence, relationships with family and friends, and engaging in exercise and social activities.
  ○ Frail older people living with CKD most commonly choose to set goals in the domains of function (mobility, exercise and activities of daily living), physical health (weight loss, risk factor modification and symptom control), and social engagement and leisure (relationships, hobbies and sport), which appear more aligned to the priorities of frail older people rather than those living with CKD.
  ○ The number and nature of set goals were similar across participant frailty status, quality of life (EQ‐5D‐5L) scores, and CKD stage, suggesting that these characteristics do not influence the priorities of frail older people with CKD.
- Why does this paper matter?
  ○ Nephrologists can seek to align their care of frail older people with CKD to these patient‐identified priorities, and given the commonality of goals between frail older people with and without CKD, there may be a role for geriatricians to contribute to the management of this group.

## 1 Introduction

Frail older people living with chronic kidney disease (CKD) have complex care needs given their multiple comorbid conditions and associated medications [1]. It is known that there is often discordance between what people living with multiple health conditions identify as primary concerns for themselves and those identified for them by their clinicians [2]. This means the bigger picture for an individual can be missed, particularly when their care is provided in a fragmented manner across different teams [3, 4]. Person‐centred care, recognized as a pillar of quality healthcare, calls on healthcare professionals to consider what matters most to an individual [4, 5]. Key to facilitating this is goal setting [3, 4]. As defined by Naik and Walling, goals in a healthcare setting are a statement of a desired health or life outcome which a person is seeking to accomplish [6]. They should be SMART (specific, measurable, achievable, relevant and timebound) and consistent with what matters most to an individual [6]. Goals indicate what is important to an individual and thus can be a reflection of their priorities [7, 8].

People living with CKD, of all ages, have highlighted kidney function, kidney failure, fatigue, mortality, life participation, and anxiety as being important to them [9]. Studies of older people have noted maintaining independence, relationships with family and friends, and engaging in exercise and social activities to be important to them [10, 11, 12]. It is unknown what older people who are both frail and living with CKD identify as their goals. By understanding this, healthcare professionals can ensure their management plans are appropriately aligned to them and deliver care that is more person‐centred.

The aims of this study are to: (1) identify and categorize the goals of frail older people living with CKD; and (2) assess the association of these goals with their frailty status, quality of life scores, and stage of CKD.

## 2 Methods

This research utilizes baseline data collected from participants in the GOAL trial, for which the protocol has been published elsewhere [13]. It was a cluster‐randomized controlled trial that sought to understand whether frail older people living with CKD who received a comprehensive geriatric assessment (CGA) were better able to attain their goals than those who received usual care only.

### 2.1 Study Design

**FIGURE 1:**
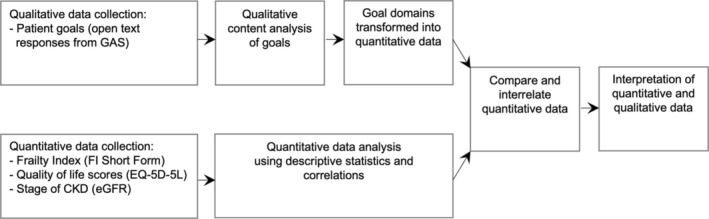
The relationship and sequence of qualitative and quantitative research components—adapted from Creswell et al. [14] GAS, Goal Attainment Scaling; FI, Frailty Index; eGFR, Estimated glomerular filtration rate).

This is a mixed methods study using a triangulation design [14]. The data transformation model variant of triangulation design was used to transform qualitative data into quantitative data, enabling analysis of these two data types in a complementary manner [14]. Figure 1, adapted from Creswell and colleagues' seminal work on mixed methods research design [14], helps to conceptualize the relationship and sequence of the study's qualitative and quantitative research components. The transformation model enabled qualitative data (goals expressed as free‐text responses) to be converted into quantitative data (frequency counts). Doing so allowed for an interpretation of the described goal categories and participant characteristics, including a person's degree of frailty, quality of life scores, and severity of CKD. The checklist for mixed‐methods research manuscript preparation provided by Lee [15], recommended by the EQUATOR Network [16], helped guide the reporting of this study.

### 2.2 Participants and Recruitment

GOAL trial participants were frail (Frailty Index [FI] of > 0.25 as measured by the FI Short Form [17]) older people (≥ 65 years, or ≥ 55 if Aboriginal and/or Torres Strait Islander [First Nation's Australian]) living with moderate to severe CKD (eGFR ≤ 59 mL/min/1.73 m^2^). For the study reported here, all GOAL trial participants who proceeded to set goals at baseline via Goal Attainment Scaling (GAS) were included. Those who did not complete the goal‐setting process, due to withdrawal from the trial or another reason, were excluded.

GAS is a method for setting and then scoring the extent to which a person's individual goals are achieved [18]. The GAS process included: identifying an individual's goal; describing their current abilities; defining possible future outcomes on a five‐point scale; and, at follow‐up, scoring their actual achievement against the articulated scale [19]. It has been applied in a variety of healthcare settings [20]. GAS has been found to be reliable [21, 22] and responsive to the detection of clinically meaningful changes in older adults [23, 24, 25].

The implementation of GAS for the GOAL trial is further explained at length elsewhere [26], with the full training package available online [27], but briefly described here. At the time of enrolment into the GOAL trial, participants were issued with a GAS patient preparation information sheet to prompt self‐reflection ahead of the goal setting conversation with research staff. This was reviewed by the trial's Consumer Advisory Board during its development. There was comprehensive training for the research staff who administered GAS with trial participants. This entailed instruction on leading a goal setting meeting and guides on troubleshooting common issues and pitfalls. Prompting questions to help brainstorm potential goals were suggested in the training and included “if you could change one thing in your life, what would that be?” and “if your friends and family were here, what would they say you should focus on?”. There were simulation scenarios undertaken within the online classroom learning, and subsequent one‐on‐one simulation exercises with the lead trainer. Rapid third‐party reviews were undertaken of the first five GAS sheets each research staff member completed with trial participants.

### 2.3 Data Collection

The goals set by participants via GAS provided qualitative data for this study. Each participant was asked to set between one and five (free‐text) goals using the GAS worksheet at a meeting with study site research staff. They were able to set their goal(s) about anything, without any constraints from pre‐determined categories or lists. In the GAS worksheet [27], the first row required general wording to be entered about the participant's goal. This was sometimes broad (e.g., “to lose weight”) and other times more specific (e.g., “through healthy eating, reduce weight to 95.0 to 100.0 kg”), depending on how the patient conceptualized it. It was this first row which constituted the qualitative data that were input verbatim by research staff into Research Electronic Data Capture (REDCap), a secure, web‐based software platform hosted at The University of Queensland [28]. The worksheet then provided further rows to articulate a five‐point outcome scale from “much worse than expected” through to “much better than expected.” It was through these outcome scales that each participant, with the assistance of the research staff, made their goal SMART (specific, measurable, achievable, relevant and timebound).

Quantitative baseline characteristics were collected from participants by research staff at each study site. Stage of CKD was based on a participant's estimated glomerular filtration rate (eGFR) calculated by the CKD‐EPI equation [29] and categorized as moderate CKD—stage 3 and 4 (eGFR: 15–59 mL/min/1.73 m^2^) and severe CKD—stage 5 and 5D (eGFR below 15 mL/min/1.73 m^2^, including those receiving dialysis). Frailty was measured by the FI Short Form, where values were between 0 and 1. A higher number indicates more severe frailty, and those with FI > 0.25 are defined as frail [30, 31, 32]. For the GOAL trial, we have categorized the FI as moderate frailty—FI of > 0.25 to < 0.36; severe frailty—FI of 0.36 to < 0.45; and very severe frailty—FI of ≥ 0.45, based on previous studies [33, 34].

EQ‐5D‐5L was used to assess a participant's quality of life. This measure considered five domains: mobility; self‐care; usual activities; pain/discomfort; and, anxiety/depression [35]. It has been found to be feasible for use in older people [36]. An index score was derived from an Australian dataset whereby the participant's level (from 1 to 5) in each domain (mobility, self‐care etc.) corresponded to a value set decrement to give a score which ranges from −0.301 to 1 [37]. A score closer to 1 denoted more favorable health. A score of 0 was ‘equivalent to death’, with values less than 0 indicating ‘worse than death’ [38]. Participants also self‐reported a health visual analogue scale (EQ‐VAS) ranked from 0 (the worst health imaginable) to 100 (the best health imaginable).

### 2.4 Data Analysis

We used deductive content analysis [39] to analyze the goals set by participants to enable a greater understanding of their identified priorities. Research staff at the sites and study investigators familiarized themselves with the data by reading the goals. At the outset of The GOAL Trial, five broad goal categories were identified by investigators to aid in the subsequent content analysis: physical health (including kidney function, symptom control and fatigue); psychological health (including mood and resilience); function (including occupational and activities of daily living); planning (including finances and advance care planning); and social engagement (including life participation). These categories were established based on the investigators' knowledge of this population from their clinical roles as geriatricians and nephrologists, along with literature about the goals and priorities of older people [40, 41, 42, 43, 44, 45] and people living with CKD [9, 46, 47]. These categories provided a matrix for the content analysis, whereby goals were coded to the categories they best corresponded to, but did not constrain what goals a trial participant could set [48].

At the time of entering a participant's goals into REDCap, the 27 research staff at the study sites simultaneously coded each goal into one of the five pre‐determined categories. When a goal related to more than one category, nurses were advised to select the primary category. As part of the trial's site activation, research staff were engaged in training on GAS (explained elsewhere [26]) where they were familiarized with the pre‐specified goal categories, underwent simulation exercises to select the appropriate category, and were instructed how to enter data into REDCap. To enhance reliability and trustworthiness [49] of the content analysis, one of the paper's authors (BL) independently coded each goal into the five categories after all data were collected. When there were conflicts between the coding decisions in the first and second round of coding, a researcher (KL) with extensive qualitative experience in health services research, was consulted to resolve disputes. During these consultations, the name of the ‘social engagement’ domain was modified to ‘social engagement and leisure’ to more appropriately reflect participants' goals. The name of the ‘planning’ domain was also modified to ‘future readiness’ to better capture the sentiments participants expressed in goals coded to that category by our coders. No other changes to the pre‐determined categories were deemed necessary through this process, with no new categories added. Descriptive accounts were produced for each category.

The goal categories applied in the content analysis were transformed into quantitative data using frequency counts so that quantitative analysis could be undertaken via descriptive analysis to examine associations between goal categories and participant characteristics. A weighted analysis of frequencies was undertaken where a participant had more than one goal (e.g., where they had one goal in the physical domain and one in the function domain they were weighted as 0.5 each). Frequency counts were expressed as numbers with percentages, and continuous variables were expressed as means with standard deviations (SD) or medians with interquartile ranges (IQR), depending on distribution characteristics. These calculations were performed using International Business Machines (IBM) Statistical Package for the Social Sciences (SPSS) for Windows, Version 29.0 [50]. Inferential statistics were not undertaken as this work was predominantly exploratory in nature.

### 2.5 Ethics Statement

The GOAL trial obtained ethical approval from the Metro South Human Research Ethics Committee (Reference: HREC/2020/QMS/62883). All trial participants provided written informed consent. The GOAL trial was prospectively registered (ClinicalTrials.gov: NCT04538157).

## 3 Results

### 3.1 Participant Characteristics

The GOAL trial enrolled 240 participants, though 16 withdrew prior to setting goals, leaving 224 included in this study. Participants were predominantly male (*n* = 125, 56%), white/European (*n* = 187, 84%), with a mean age of 77 years (SD: 6.7). There were 119 participants (53%) who had moderate CKD (stage 3 or 4) and 105 participants (47%) who had severe CKD (stage 5 and 5D). The cohort's median FI of 0.39 (IQR: 0.33–0.47) sat within the severe frailty category. The median EQ‐5D‐5L quality of life score was 0.83 (IQR: 0.63–0.92). The range in our cohort was −0.133 to 1. The median visual analogue scale (EQ‐VAS) was 60 out of 100 (IQR: 50–75). Table 1 presents the participants' characteristics.

**TABLE 1:** Participant characteristics.

Characteristic	Total (*n* = 224)
Age, years—mean (SD)	77 (6.7)
Males	125 (55.8)
Females	99 (44.2)
Ethnicity	
White/European	187 (83.5)
Australian First Nations	8 (3.6)
Asian	9 (4)
Black/African/Caribbean	0 (0)
Other	19 (8.5)
Unknown	1 (0.4)
CKD stage	
Stage 3	54 (24.1)
Stage 4	65 (29)
Stage 5	28 (12.5)
Stage 5D	77 (34.4)
Frailty index—median (IQR)	0.39 (0.33–0.47)
EQ‐5D‐5L index score—median (IQR)	0.83 (0.63–0.92)
EQ‐5D‐5L VAS—median (IQR)	60 (50–75)
Number of goals set	
1	88 (39.3)
2	95 (42.4)
3	35 (15.6)
4	5 (2.2)
5	1 (0.4)
Goals by domain—count of all goals (*n* = 408)	
Function	172 (42.2)
Physical health	86 (21.1)
Social engagement and leisure	81 (19.9)
Psychological health	58 (14.2)
Future readiness	11 (2.7)
Goals by domain—weighted by participant (*n* = 224)^a^	
Function	103.3 (46.1)
Physical health	44.3 (19.8)
Social engagement and leisure	42 (18.8)
Psychological health	29.6 (13.2)
Future readiness	4.8 (2.2)

*Note*: Unless indicated, results presented as: number (percentage).

Abbreviations: CKD, Chronic Kidney Disease; EQ‐VAS, EQ‐5D‐5L Visual Analogue Scale; FI, Frailty Index; IQR, Interquartile Range; SD, Standard Deviation.

^a^ Weighted analysis of frequencies (e.g., where they had one goal in the physical health domain and one in the future readiness domain they were weighted as 0.5 each).

### 3.2 Goals of Participants

Participants set 1–5 goals (408 goals in total), with most setting one (*n* = 88, 39%) or two (*n* = 95, 42%) goals. Most commonly, these goals were related to function (*n* = 172, 42%), physical health (*n* = 86, 21%), and social engagement and leisure (*n* = 81, 19.9%), and less frequently related to psychological health (*n* = 58, 14%) and future readiness (*n* = 11, 2.7%). As shown in Table 1, the weighted analysis of frequencies provided similar results.

The majority of the 172 goals set in the function category related to mobility (*n* = 104). Of those, 81 were about walking distance (e.g., “increase walking distance”), walking duration (e.g., “would like to walk for 15 min without stopping”), and walking frequency (e.g., “walk on a regular weekly basis”). The remaining 23 mobility goals covered balance (e.g., “improve confidence with balance ‐ score out of 10”) and mobility aids (e.g., “to reduce time taken to apply left lower limb prosthesis”). Forty‐two functional goals were related to other forms of exercise and covered modes (e.g., “attend hydrotherapy”), duration (e.g., “I want to extend the length of time I exercise”), frequency (e.g., “to do prescribed strength and balance exercise more times per fortnight”), and outcomes of exercise (e.g., “to increase muscle strength and be able to get off the floor after exercising”). There were 26 goals related to activities of daily living, encompassing community access (e.g., “taking the bus to the nearby shopping Centre”), personal activities (e.g., “wash and dress independently without assistance”) and domestic duties (e.g., “to cook own meals more”).

In the physical health category, 38 of the 86 goals considered healthy lifestyle factors. The majority of these (*n* = 26) related to weight loss (e.g., “to lose up to 2 kg”), with other goals concerning various cardiovascular risk factors (e.g., “quit smoking”). The control of symptoms was important to some participants, with 24 goals set about managing pain (e.g., “to reduce frequency of neuropathic pain episodes per week”) or other symptoms (e.g., “to ease severity of an itchy body”). There were eight goals about medications, regarding polypharmacy (e.g., “to decrease number of medications”) and adherence (e.g., “to take EPO injection as prescribed”). Goals were also set around biochemical markers, i.e., blood tests, (e.g., “to optimize potassium level”) and engagement with healthcare providers (e.g., “attend specialist appointments to improve vision”). Four goals related to avoiding dialysis (e.g., “to not end up on dialysis”).

The 82 goals in the social engagement and leisure category reflected a range of interests and priorities for participants. Most (*n* = 22) were about relationships, including family (e.g., “to see sisters in person more frequently per week”), friends (e.g., “to be able to have a friend for conversation at the Residential Aged Care Facility”), and romantic (e.g., “increase outings with girlfriend to at least 2× weekly”). Sixteen were about general socialization (e.g., “increase number of social outings per month”). Participants noted a variety of artistic pursuits (e.g., “to book and attend an art class again”) and craft and hobby interests (e.g., “to get model train set up and running”), as well as sports (e.g., “to practice golf swing more than once per week”) and gardening (e.g., “to weed and plant first section of side garden”). Travel was the content of eight goals (e.g., “travel in motorhome 1 in every 4 weeks”).

Participants set 58 goals in the psychological health category. Most of these (*n* = 40) related to matters of sleep, including its initiation (e.g., “reduce length of time taken to fall asleep” and “fall asleep at an earlier time”), duration (e.g., “increase sleep duration at night”), quality (e.g., “reduce the number of times I wake up at night”), and daytime somnolence (e.g., “reduce the need to sleep in the afternoon”). Nine goals related to mood and anxiety, with some participants wanting to reduce mental health symptoms (e.g., “experience less depressive episodes – ‘under doona days’–per fortnight” and “reduce weekly frequency of anxiety episodes about falling”). The five goals related to cognition all focused on memory, including improvements to short‐term memory (e.g., “retain information after viewing episode of TV series”) and the use of memory aids (e.g., “record healthcare visits in diary to improve memory”). Other goals categories in the psychological health category related to confidence (e.g., “walk with confidence to cross main road”) and satisfaction (e.g., “improve satisfaction with my career skills”).

**FIGURE 2:**
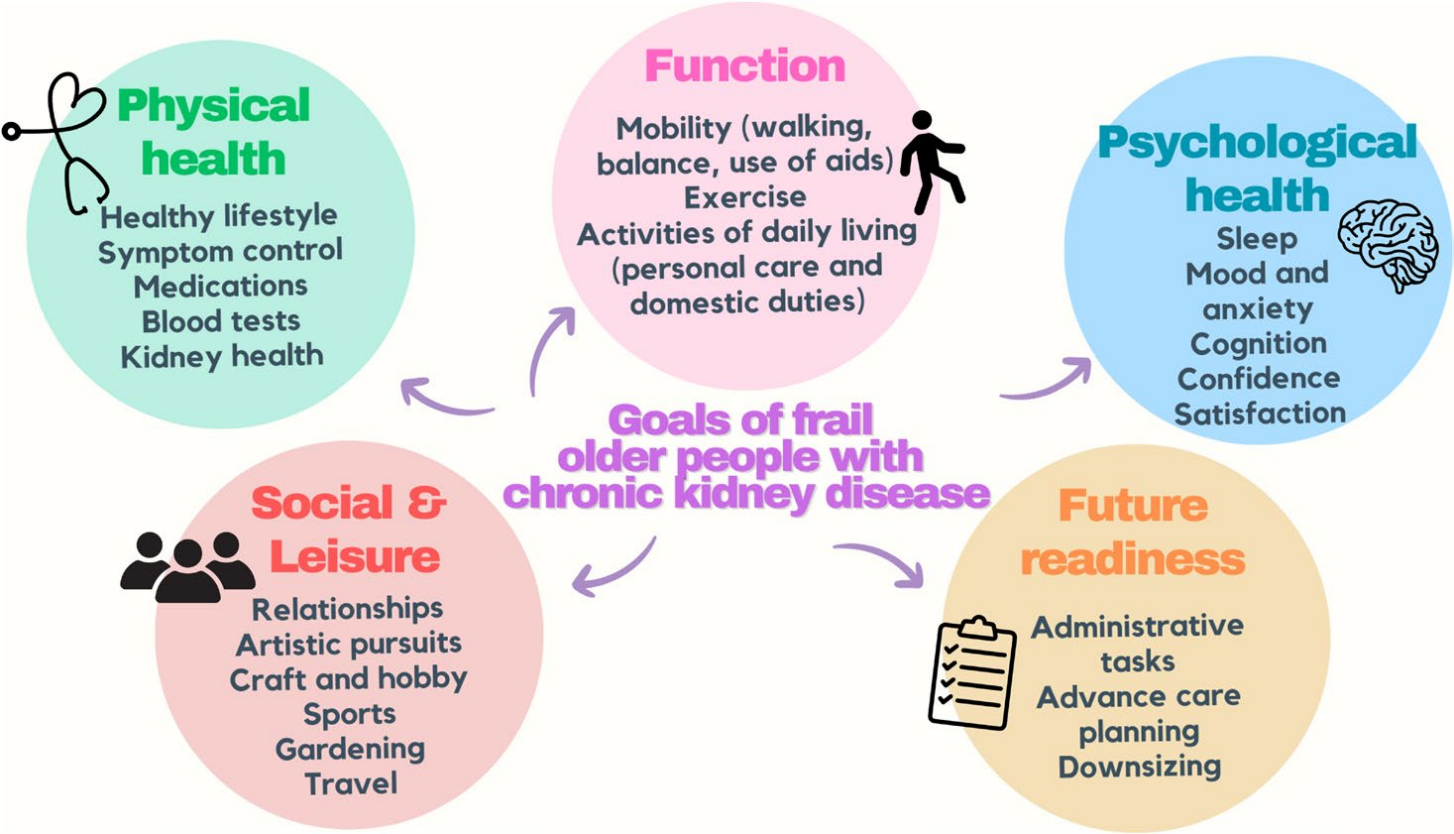
Goals of frail older people living with moderate to severe chronic kidney disease.

Of the 11 future readiness category goals, four goals focused on general administrative tasks such as government paperwork (e.g., “to get another referral to social worker so he can get help with sorting ATO {taxation} and Centrelink {pension} paperwork”). Other goals related to advance care planning tasks (e.g., “organize paperwork in preparation of funeral planning” and “re do Will and EPOA {enduring power of attorney, the formal appointment of a substitute decision‐maker}”) and to downsizing their home (e.g., “distribute possessions in readiness for downsizing to a smaller home”). Figure 2 provides a visual representation of the descriptive accounts.

### 3.3 Goal Categories and Participant Characteristics

Participant characteristics were described by the number of goals a person set, and are presented in Table 2. They were also described by the domain in which they were set, as presented in Table 3. There were 146 unique participants who had a goal in the function category, 76 in physical health, 66 in social engagement and leisure, 50 in psychological health, and nine in future readiness. Fifty‐five participants set two or more goals in the same category. Their characteristics were only used once. The age, sex, CKD severity, frailty status, and quality of life scores between these participant cohorts were similar. There were also similarities between participant characteristics when grouped by the number of goals they each set, as displayed in Table 2.

**TABLE 2:** Participant characteristics by number of goals set.

Characteristic	Participant set 1 goal *n* = 88	Participant set 2 goals *n* = 95	Participant set 3/+ goals *n* = 41
Age, years—median (IQR)	77 (73.3–83.8)	75.7 (71.5–81.5)	76.5 (70.3–80.6)
Males	48 (54.5)	52 (54.7)	25 (61)
Females	40 (45.4)	43 (45.3)	16 (39)
CKD severity			
Moderate CKD (stage 3 + 4)	50 (56.8)	43 (45.3)	26 (63.4)
Severe CKD (stage 5 + 5D)	38 (43.2)	52 (54.7)	15 (36.6)
Frailty index–median (IQR)	0.37 (0.31–0.46)	0.40 (0.35–0.49)	0.39 (0.33–0.46)
EQ‐5D‐5L index score—median (IQR)^a^	0.83 (0.64–0.92)	0.82 (0.56–0.89)	0.86 (0.74–0.93)
EQ‐5D‐5L VAS—median (IQR)^a^	60 (50–75)	60 (50–75)	60 (50–70)

Abbreviations: CKD, Chronic Kidney Disease; EQ‐VAS, EQ‐5D‐5L Visual Analogue Scale; FI, Frailty Index; IQR, Interquartile Range; SD, Standard Deviation.

^a^ Denotes that two participants did not have an EQ‐5D‐5L completed, and thus this missing data was excluded.

**TABLE 3:** Participant characteristics by goal domain.

Characteristic	Function (*n* = 146^a^)	Physical health (*n* = 76^a^)	Social engagement and leisure (*n* = 66^a^)	Psychological health (*n* = 50^a^)	Future readiness (*n* = 9^a^)
Age, years—median (IQR)	75.7 (71.4–82.1)	75.7 (71.8–81.4)	76.8 (72.8–80.8)	76.9 (70.3–82.9)	77 (69.1–78.7)
Males	74 (50.7)	46 (60.5)	39 (59.1)	29 (58)	6 (66.7)
Females	72 (49.3)	30 (39.5)	27 (40.9)	21 (42)	3 (33.3)
CKD severity					
Moderate CKD (stage 3 + 4)	78 (53.4)	44 (57.9)	32 (48.5)	25 (50)	6 (53.3)
Severe CKD (stage 5 + 5D)	68 (46.6)	32 (42.1)	34 (51.5)	25 (50)	3 (46.7)
Frailty index—median (IQR)	0.40 (0.33–0.47)	0.38 (0.33–0.44)	0.38 (0.31–0.45)	0.41 (0.34–0.47)	0.38 (0.30–0.48)
EQ‐5D‐5L index score—median (IQR)^b^	0.82 (0.62–0.92)	0.85 (0.66–0.92)	0.85 (0.66–0.92)	0.82 (0.58–0.90)	0.85 (0.50–0.92)
EQ‐5D‐5L VAS—median (IQR)^b^	60 (50–75)	62.5 (50–75)	57.5 (50–75)	60 (43.8–75)	60.0 (43.8–75)

Abbreviations: CKD, Chronic Kidney Disease; EQ‐VAS, EQ‐5D‐5L Visual Analogue Scale; FI, Frailty Index; IQR, Interquartile Range; SD, Standard Deviation.

^a^ Where a participant set two or more goals in the same domain, their participant characteristics were used only once.

^b^ Denotes that two participants did not have an EQ‐5D‐5L completed, and thus this missing data was excluded.

## 4 Discussion

This study reports the goals of a group of frail older people living with CKD. Participants most frequently set goals related to function (mobility, exercise and activities of daily living), physical health (weight loss, risk factor modification and symptom control) and social engagement and leisure (relationships, hobbies and sport). Participants' goals provided an insight into what matters most to them and may help in understanding what the priorities might be for other frail older people living with CKD. The characteristics of participants were similar when describing frailty status, quality of life scores, and stage of CKD by the number of goals set and the domains in which goals were set.

To our knowledge, this is the largest cohort of frail older people living with CKD who have had their goals elicited and categorized. This study is congruent with the “The 4 Ms” set out in the Institute for Healthcare Improvement's guidance for creating age‐friendly health systems: what matters; medication; mobility; and, mentation [51]. Primarily, it allows a better understanding of “what matters” for this population. By knowing their goals, healthcare providers can then be better positioned to tailor their system design and care plans to what frail older people with CKD have identified as being important to them. The other components of age‐friendly health systems (medication, mobility, and mentation) are well represented in the subcategories which emerged in documenting what goals this population set. This suggests that the institute's vision for an ideal health system design for older adults aligns with the goals prioritized by frail older people with CKD.

The high frequency with which goals were set in the domains of function, physical health, social engagement, and leisure broadly reflects the themes that were reported in prior interviews Hall and colleagues conducted. Their work identified that for 12 older people (mean age of 81) receiving hemodialysis, who were often frail (50%), what most mattered to their quality of life was physical well‐being, including retaining independence for activities of daily living and having symptom control, as well as social support, including practical assistance and socialization [52].

Our results also share similarities with studies of frail older people more generally, whose CKD status is not reported. Robben et al. completed a retrospective chart audit of 162 goals set by 140 community dwelling older people (all aged > 70 years; all frail) which showed goals were most frequently set in relation to health problems, living accommodation, social and family relationships, and mobility [12]. Two recent Dutch studies asked frail older people to choose from a predetermined short list of priorities. Concordant with the themes of our study, Festen and colleagues' cohort of older people (*n* = 350; median age 78.5 years; 59.7% frail) most commonly chose “maintaining independence” as their health outcome priority from a total of four options [53]. Van der Klei and colleagues found older people (*n* = 1278; median age 76 years; 44% frail) weighted “preventing nursing home admission” and “staying independent” as the two most important goals from a menu of seven options [54]. The results for our population are also consistent with goals and priorities elicited through interviews with older people for whom frailty status was not reported [10, 11, 55].

van der Klei et al. stratified their results by frailty status and noted no associations between a participant's frailty status and stated preferences [54]. This consistency across older people of varied frailty status was congruent with our findings that the characteristics of participants, including frailty status, were similar between the five goal categories. This suggests that the goals of older people are not necessarily influenced by their frailty status.

Our results did diverge from prior literature when comparing them to the stated priorities of other, younger people living with CKD. Gonzalez and colleagues undertook focus groups with 54 people living with CKD and 13 caregivers of people living with CKD (70% aged 60 or younger; no frailty status reported) which noted their priorities to be kidney function, kidney failure, fatigue, mortality, and life participation [9]. While life participation was similar to GOAL trial participant goals, the other priorities were not commonly expressed. This may indicate that, for those living with CKD, the primary concerns of older people are distinct from those of younger people and more in keeping with older people generally. This is a new finding.

The outcomes of our study have implications for nephrologists in their management of frail older people with CKD. They should be mindful that what is important to older people differs from the priorities of younger people, irrespective of a shared CKD diagnosis. Given the commonality of findings between frail older people with and without CKD, geriatricians may have a role in contributing to the management of this group. Whether a review by a geriatrician better enables frail older people to attain their goals will be better understood when the results are reported of the GOAL trial, the cluster randomized controlled trial from which these data come [13]. Our findings can also guide researchers working with frail older people living with CKD to select study outcomes that most align with their person‐centred goals. Future research could consider how best to practically operationalize consideration of an individual's goals in a meaningful and routine manner.

### 4.1 Strengths and Limitations

Our study contributes new knowledge as we sought to understand the priorities of frail older people who are also living with CKD, whereas past research has generally looked at participants with just one of these attributes. The participant sample size was a strength of this research. Our cohort of 224 participants is relatively substantial when compared to past qualitative and mixed‐methods research that has been undertaken to understand the goals of frail people, older people, or those living with CKD. Our use of GAS for goal setting was supported by robust training of the healthcare professionals administering it to participants, detailed elsewhere [26], which ensured rigor in its implementation. Additionally, unlike some previous research [53, 54], participants were able to set goals about anything and were not limited to selecting from pre‐specified priorities.

The primary limitation of this research was the constraints that the GAS process imposed on goal setting. As the GOAL trial's primary outcome was GAS attainment at 3 months, participants may have moderated their priorities to fit this timeframe and been disinclined to identify something with a longer duration, such as avoiding CKD progression to dialysis. To minimize the impact of this, research staff were able to work with patients to identify short‐term goals that related to longer‐term goals. Goals for GAS have to be specific, measurable, and have five potential outcomes prespecified. As a result, participants may have chosen a priority area for which an objective measure was easily recognized and understood. This may have biased participants to set goals about activities such as walking (measured by distance in meters, or duration in minutes) and managing their weight (measured by loss in kilograms), rather than a significant concern with a less clear metric such as managing fatigue or feeling less dependent on loved ones. This may have accounted, to some extent, for why there was a greater proportion of goals related to function than seen in prior studies where there was a more equal representation of goals across function, leisure, and social connection domains [9, 11]. Potentially, the advanced disease state of our participants may have also contributed to this observed difference, given that Shwayder and colleagues [56] similarly found an emphasis on function goals in their population of people living with advanced coronary artery disease.

Challenges with coding goals to one of the five categories presented another limitation of this research. Firstly, while goals were coded into one primary category, some potentially fit in two or more. As an example, “to walk around the block with wife” was coded in the function category, but may have also been of relevance to the social engagement and leisure domain. Overlapping goals across categories may indicate a combination of multiple health values [57]. In some instances, the decision about a whole group of goals, notably those in relation to sleep, could have arguably been allocated to multiple domains. The decision taken to place sleep in the psychological health category by our researchers, based on our content analysis, may vary as to how others might have handled it. Secondly, the role of the first coder was completed by many different research staff over the 15 study sites the GOAL trial recruited participants at. This introduced greater variability, and potentially inconsistencies, in coding decisions. Finally, the second and third coders did not benefit from the contextual knowledge the first coder had from being present for discussions with the participant at the time goals were set. To address some of these challenges, standardized training [26] was provided at the outset of the trial to research staff which included third‐party reviews of the initial GAS worksheets they completed with participants. There were also comprehensive discussions held between the second and third coders to ensure the appropriateness of the final coding decision.

Research into patient goals has been relatively minimal to date. Prior work examining younger populations generally does not report their frailty status, so comparisons have to be interpreted with some caution. Future research into the goals of frail older people may wish to consider coding to a taxonomy of goal types (i.e., behavior goal or an outcome goal etc.), exploring whether there are differences between goal domains, and whether certain participant characteristics influence the types of goals participants set.

### 4.2 Conclusion

This study sought to detail and categorize the identified goals of a cohort of frail older people living with CKD, and describe a person's frailty status, quality of life scores, and stage of CKD by the number of goals and the domains in which they were set. Participants' characteristics and the goals they set by GAS were analyzed via a mixed‐methods study utilizing a triangulation design and data transformation model. This research provides new knowledge about what frail older people living with CKD find important and seek to prioritize when asked to set goals achievable in 3 months. Results highlighted the value participants place on function, physical health, social engagement, and leisure. The number and nature of set goals were similar across participant frailty status, quality of life scores, and CKD stage, suggesting that these characteristics do not influence the priorities of frail older people with CKD. Our findings can help guide healthcare professionals in ensuring management plans consider the identified priorities for this population. Given this group's goals appear to be more aligned with their frail older peers, rather than other people living with CKD, it prompts consideration of the role geriatricians may contribute to the care of this population.

## Author Contributions

B.L., K.L., E.M.P., A.K.V., L.E.H., and R.E.H. were involved in this study's design and concept. Data was acquired by the GOAL trial and overseen by the Trial Steering Committee and the Trial Management Committee, of which B.L., E.M.P., A.K.V., D.W.J., C.M.H., L.E.H., C.K., M.M., A.J., and R.E.H. are current or past members. R.E.H. is the Principal Chief Investigator of the GOAL Trial. B.L., K.L., and E.M.P. completed the primary data analysis and interpretation. B.L. led the writing of the manuscript with input from K.L., E.M.P., A.K.V., D.W.J., C.M.H., L.E.H., C.K., M.M., A.J., and R.E.H. All authors contributed to revising the manuscript, and they read and approved the final version.

## Conflicts of Interest

Andrea K. Viecelli receives grant support from a Queensland Advancing Clinical Research Fellowship and an NHMRC Emerging Leader Grant (APP1196033). David W. Johnson has received consultancy fees, research grants, speakers honoraria and travel sponsorships from Baxter Healthcare and Fresenius Medical Care, consultancy fees from Astra Zeneca, Bayer, and AWAK, speaker's honoraria from ONO and Boehringer Ingelheim & Lilly, and travel sponsorships from Ono and Amgen. He is a current recipient of an Australian National Health and Medical Research Council Leadership Investigator Grant (APP1194485). Carmel M. Hawley has received fees paid to her institution from Janssen and GlaxoSmithKline; Advisory Board fees paid to her from Otsuka; Research Grants to her institution from Otsuka, Shire, Fresenius, and Baxter. In addition, she has received grants paid to her institution from the Polycystic Kidney Disease Foundation of Australia. None of these is related to the current study. All other authors declare no financial, personal, or potential conflicts to disclose.

## Sponsor's Role

The University of Queensland is the sponsor for the GOAL Trial. The Australasian Kidney Trials Network (AKTN) at the University of Queensland is the coordinating centre for the trial and is involved in overall study activities, including study design, collection, management, analysis, and interpretation of data, writing of the report, and decision to submit the report for publication. This research is funded by a National Health and Medical Research Council grant. Specifically, a Targeted Call for Research into Frailty in Hospital Care was awarded in 2019 (APP1178519). The funder has no role in study design, data collection, analysis, or publication decisions.
